# Supplementary material for: Volumetric extrusive rates of silicic supereruptions from the Afro-Arabian large igneous province
Source: Nat Commun. 2021 Nov 2;12:6299. doi: 10.1038/s41467-021-26468-5 (PMC8563981; doi:10.1038/s41467-021-26468-5)
Supplement: Supplementary file 1 — Supplementary Information [file 41467_2021_26468_MOESM1_ESM.pdf]

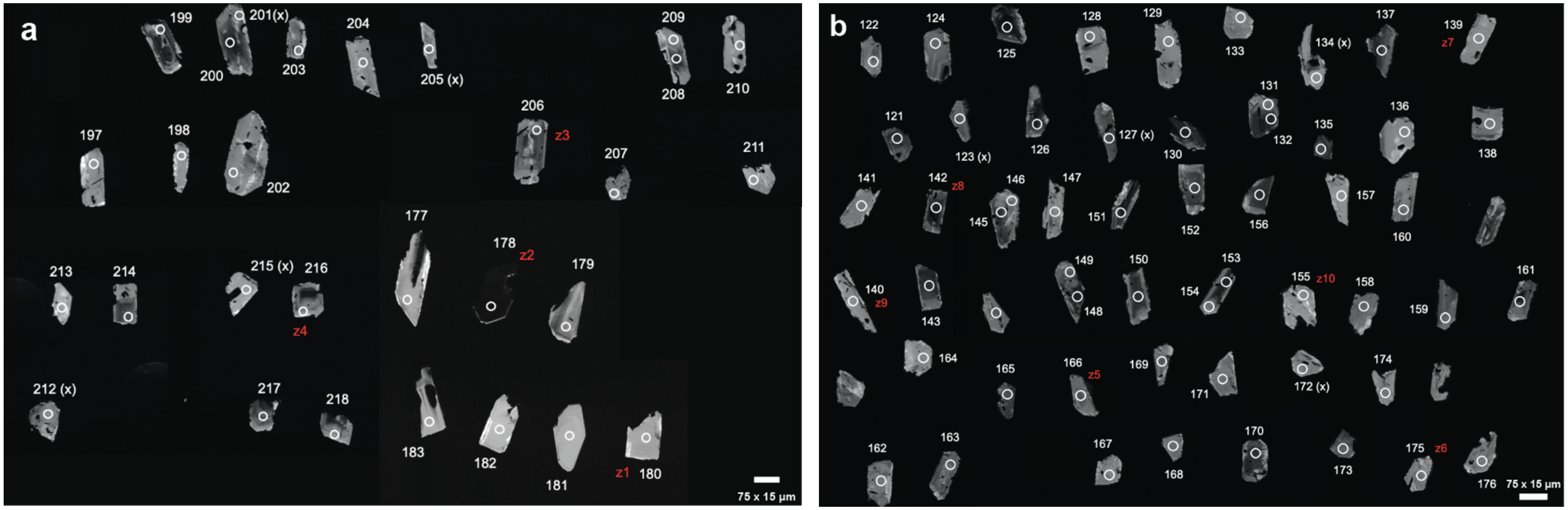

Supplementary Fig. 1. Composite cathodoluminescence (CL) images of zircon from Escarpment Ignimbrite sample YIU99026 taken at 150x (subset a) and 200x (subset b) magnification. Circles are labelled by laser ablation inductively coupled mass spectrometry (LA-ICP-MS) spot analysis (Supplementary Data 1). Zircon selected for ID-TIMS dating are denoted by ‘zX’ (Supplementary Data 1). LA spots that either burned through the crystal during analysis or raw data spectrum that indicated inclusions are denoted by ‘(x)’ and are not included Supplementary Data 1.

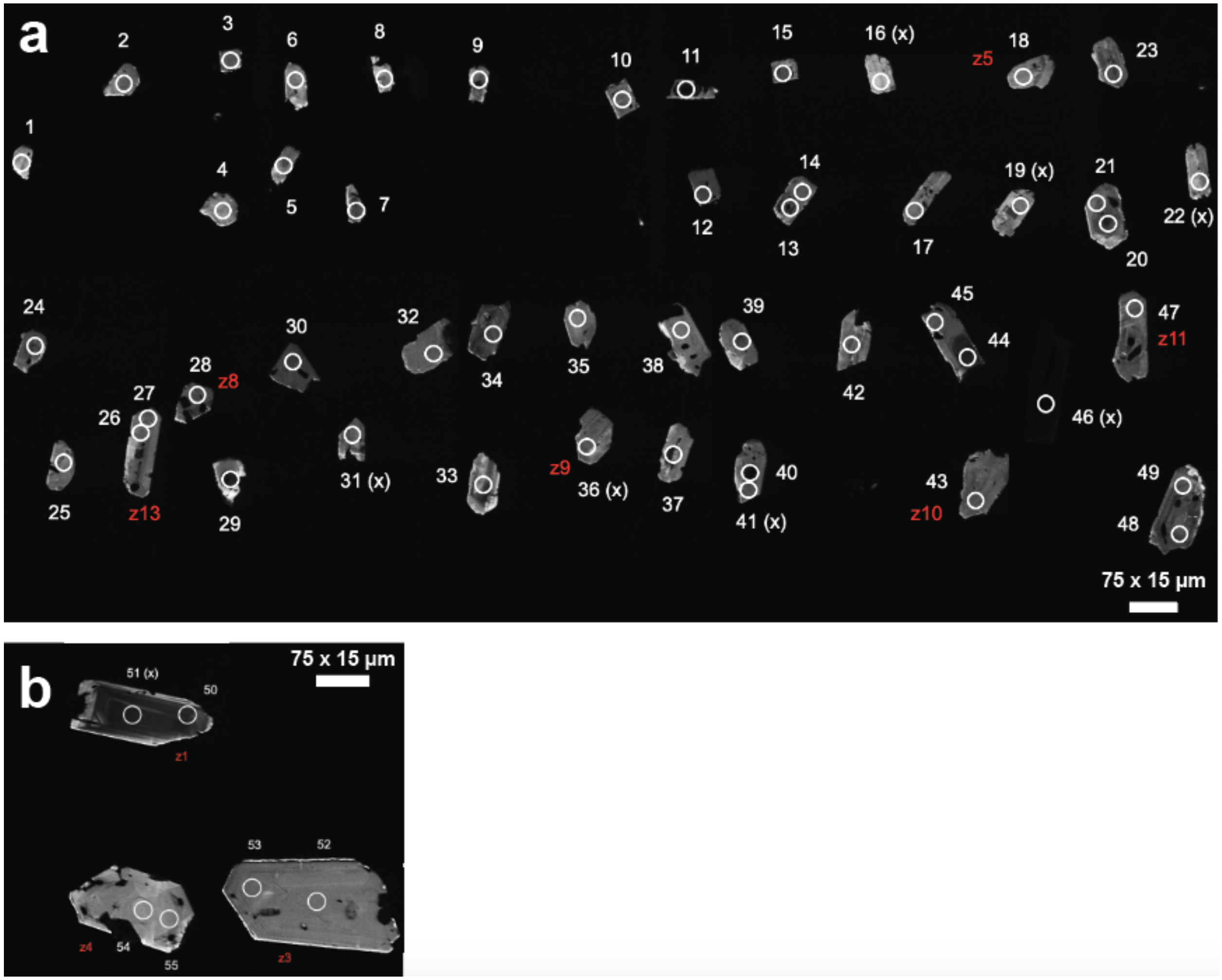

Supplementary Fig. 2. CL images of zircon from SAM Ignimbrite sample YIU99210 taken at 200x (subset a) and 350x (subset b) magnification. Circles are labelled by LA-ICP-MS spot analysis (Supplementary Data 1). Zircon selected for ID-TIMS dating are denoted by ‘zX’ (Supplementary Data 1). LA spots that either burned through the crystal during analysis or raw data spectrum that indicated inclusions are denoted by ‘(x)’ and are not included Supplementary Data 1.

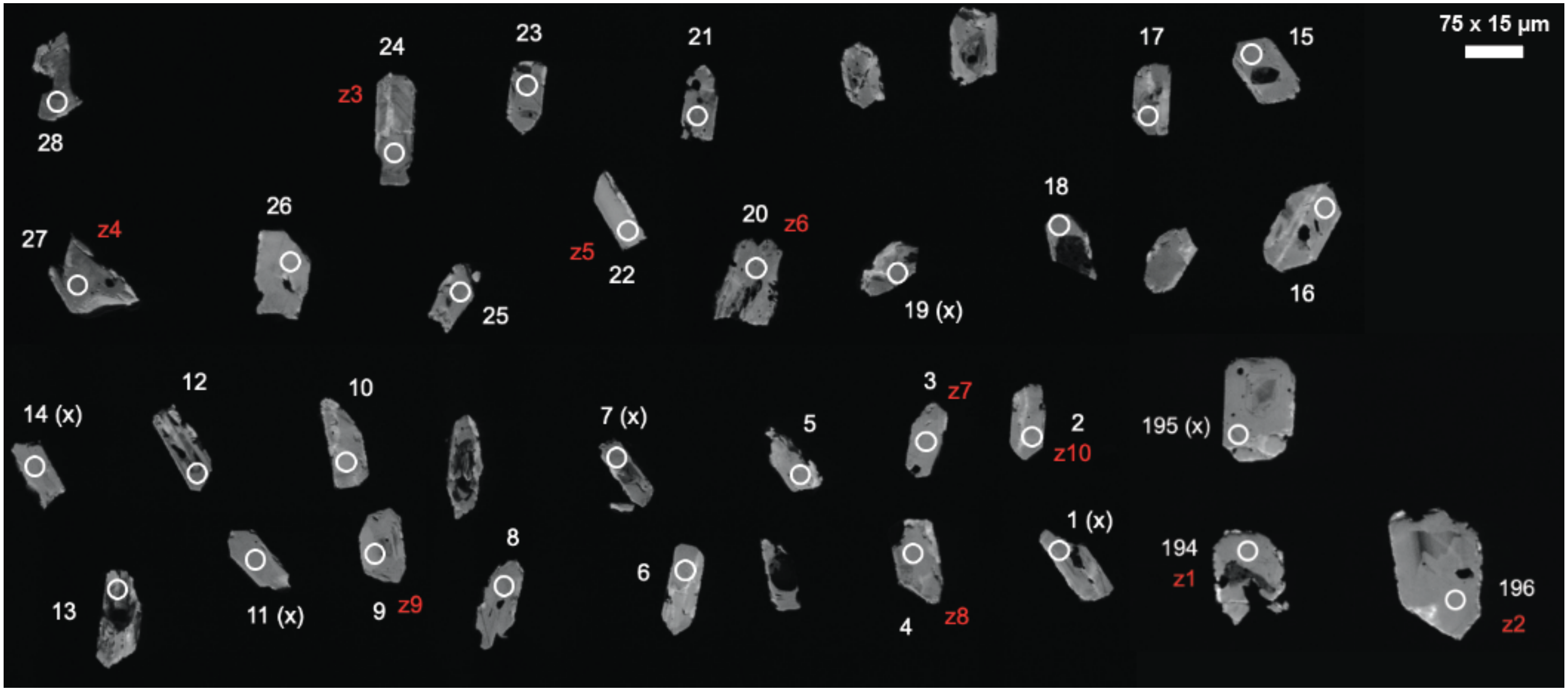

Supplementary Fig. 3. CL images of zircon from Sana'a Ignimbrite sample YIU99206 taken at 200x magnification. Circles are labelled by LA-ICP-MS spot analysis (Supplementary Data 1). Zircon selected for ID-TIMS dating are denoted by 'zX' (Supplementary Data 1). LA spots that either burned through the crystal during analysis or raw data spectrum that indicated inclusions are denoted by '(x)' and are not included Supplementary Data 1.

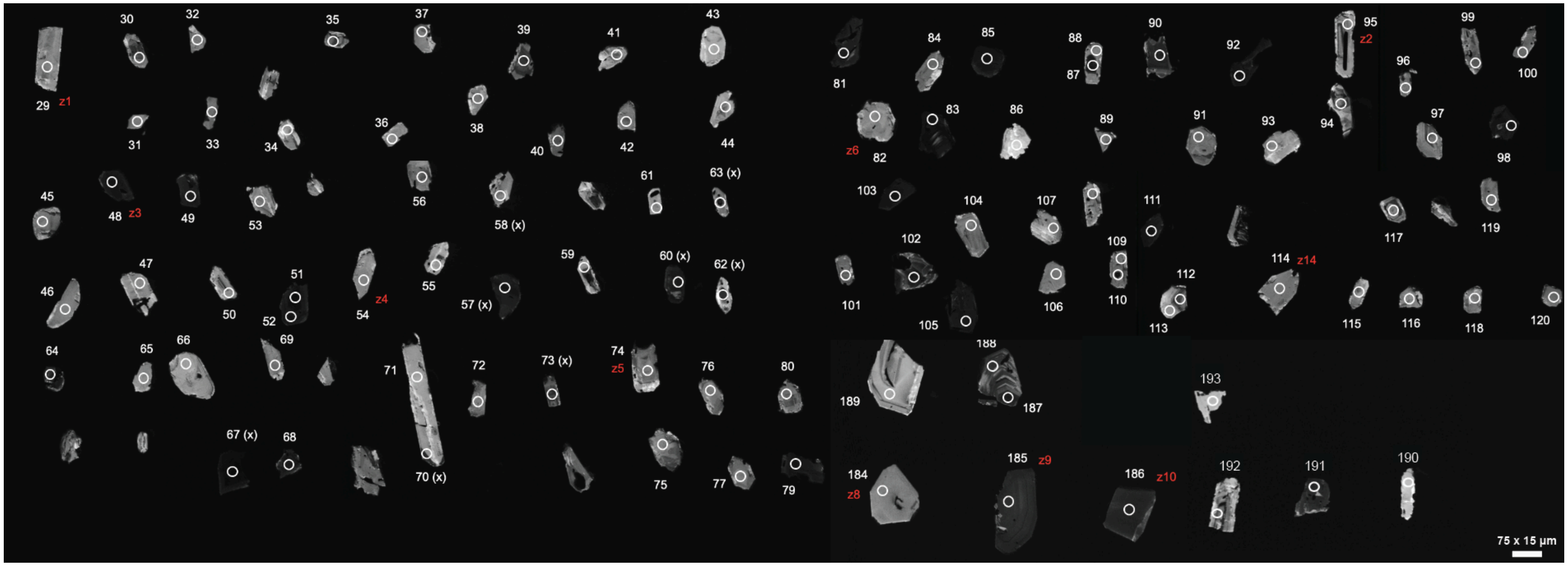

Supplementary Fig. 4. CL images of zircon from Iftar Alkalb sample YIU99172 taken at 200x magnification. Circles are labelled by LA-ICP-MS spot analysis (Supplementary Data 1). Zircon selected for ID-TIMS dating are denoted by ‘zX’ (Supplementary Data 1). LA spots that either burned through the crystal during analysis or raw data spectrum that indicated inclusions are denoted by ‘(x)’ and are not included Supplementary Data 1.

$^{40}Ar/^{39}Ar$ ages were calculated using a 28.201 ± 0.046 Ma monitor age for the Fish Canyon Tuff sanidine[1]. Previous $^{40}Ar/^{39}Ar$ dates[2-4] used a 28.02 ± 0.28 Ma monitor age for the Fish Canyon Tuff sanidine. Ages were calculated used the following decay constants: $\lambda(^{40}K)_e$ = 5.810 x 10^{-11} ± 3.4 x10^{-12} yr^{-1} (value from ref. [5] and errors ref. [6], $\lambda(^{40}K)_b$ = 4.962 x 10^{-10} ± 1.7 x 10^{-11} yr^{-1} (ref. [7]), yielding $\lambda(^{40}K)_{tot}$ = 5.543 x 10^{-10} ± 1.73 x 10^{-11} yr^{-1}. $^{40}Ar/^{39}Ar$ age recalculations (Table A.1) were made using a simplified Eq. 2 from Mercer and Hodges[8]:

$$t = \frac{1}{\lambda}\ln\left[\frac{e^{\lambda t_m - 1}}{e^{\lambda t_{m_0} - 1}} \times \left(e^{\lambda t_0} - 1\right) + 1\right] \quad \text{Supplemental Equation (1)}$$

where t is the recalculated $^{40}Ar/^{39}$ age, λ is $\lambda(^{40}K)_{tot}$ = 5.543 x 10^{-10}, t_m is the new monitor age, t_{m0} is the legacy monitor age, t_0 is the legacy $^{40}Ar/^{39}Ar$ age, using the same decay constant (λ) to calculate the legacy and new $^{40}Ar/^{39}Ar$ ages. Uncertainty propagation used Eq. B.2 and B.3a – B.3g from Mercer and Hodges[8].

Sample	Unit	Legacy $^{40}Ar/^{39}Ar$ age (Ma)	± (Ma)	New $^{40}Ar/^{39}Ar$ age (Ma)	α	$\partial t/t_0$	η'	$\partial t/\lambda$	γ	$\partial t/t_{m0}$	γ'	$\partial t/t_m$	$\sigma\ t$ (Ma)
				Eq. 2	Eq. B3a	Eq. B3a	Eq. B3c	Eq. B3d	Eq. B3d	Eq. B3d	Eq. B3e	Eq. B3e	Eq. B2
YJB99.87	Bayt Mawjan Ig.	27.67 (1)	0.12	27.85	1.00	1.00	9.80×10^{-6}	3.96×10^{8}	2.42×10^{-10}	-1.00×10^{-6}	9.80×10^{-6}	9.95×10^{-6}	0.12
BM1	Ig.	28.00 (2)	0.10	28.18	1.00	1.00	9.92×10^{-7}	4.01×10^{8}	2.45×10^{-10}	-1.01×10^{-6}	9.92×10^{-6}	1.01×10^{-6}	0.10
BM5	Tuff	28.40 (2)	0.14	28.58	1.00	1.00	1.01×10^{-6}	4.07×10^{8}	2.48×10^{-10}	-1.03×10^{-6}	1.01×10^{-6}	1.02×10^{-6}	0.14
YJB88.79	Iftar Alkalb	29.48 (1)	0.08	29.67	1.00	1.00	1.04×10^{-6}	4.22×10^{8}	2.57×10^{-10}	-1.07×10^{-6}	1.04×10^{-6}	1.06×10^{-6}	0.08
YJB99.76	Green Tuff	29.59 (2)	0.12	29.78	1.00	1.00	1.05×10^{-6}	4.24×10^{8}	2.58×10^{-10}	-1.07×10^{-6}	1.05×10^{-6}	1.06×10^{-6}	0.12
YJB99.72	Akraban Andesite	29.61 (2)	0.08	29.80	1.00	1.00	1.05×10^{-6}	4.24×10^{8}	2.59×10^{-10}	-1.07×10^{-6}	1.05×10^{-6}	1.07×10^{-6}	0.08
YJB99.13	Kura'a basalt	30.03 (2)	0.26	30.22	1.00	1.00	1.06×10^{-6}	4.30×10^{8}	2.62×10^{-10}	-1.09×10^{-6}	1.06×10^{-6}	1.08×10^{-6}	0.26
EIU99-035	Shibam Kawkabam Ig.	30.16 (3)	0.13	30.35	1.00	1.00	1.07×10^{-6}	4.32×10^{8}	2.63×10^{-10}	-1.09×10^{-6}	1.07×10^{-6}	1.08×10^{-6}	0.13

Supplementary Table 1. Details on the $^{40}Ar/^{39}Ar$ age uncertainty propagation (2σ). Legacy $^{40}Ar/^{39}Ar$ ages are from (1) Riisager et al.[2], (2) Baker et al.[4], and (3) Ukstins et al.[3]. Equations are from Mercer and Hodges[8].

References

1. Kuiper, K. F. et al. Synchronizing Rock Clocks of Earth History. *Science* **320**, 500–504 (2008).
2. Riisager, P. et al. Paleomagnetism and $^{40}Ar/^{39}Ar$ Geochronology of Yemeni Oligocene volcanics: Implications for timing and duration of Afro-Arabian traps and geometry of the Oligocene paleomagnetic field. *Earth Planet. Sci. Lett.* **237**, 647–672 (2005).
3. Ukstins, I. A. et al. Matching conjugate volcanic rifted margins: $^{40}Ar/^{39}Ar$ chrono-stratigraphy of pre- and syn-rift bimodal flood volcanism in Ethiopia and Yemen. *Earth Planet. Sci. Lett.* **198**, 289–306 (2002).
4. Baker, J., Snee, L. & Menzies, M. A brief Oligocene period of flood volcanism in Yemen: implications for the duration and rate of continental flood volcanism at the Afro-Arabian triple junction. *Earth Planet. Sci. Lett.* **138**, 39–55 (1996).
5. Steiger, R. H. & Jäger, E. Subcommission on geochronology: convention on the use of decay constants in go- and cosmochronology. *Earth Plant. Sci. Lett.* **36**, 359-362 (1977).
6. Min, K., Mundil, R., Renne, P. R. & Ludwig, K. R. A test for systematic errors in $^{40}Ar/^{39}Ar$ geochronology through comparison with U/Pb analysis of 1.1-Ga rhyolite. *Geochim. Cosmochim. Acta* **64**, 73-98 (2000).
7. Audi, G., Bersillon, O., Blachot, J. & Wapstra, A. H. The NUBASE evaluation of nuclear and decay properties. *Nuclear Physics A* **729**, 3-128 (2003).

8. Mercer, C. M. & Hidges, K. V. *ArAR* – a software tool to promote the robust comparison of K-Ar and $^{40}Ar/^{39}Ar$ dates published using different decay, isotopic, and monitor-age parameters. *Chem. Geol.* **440**, 148-163 (2016).
