# Supplementary material for: Volumetric extrusive rates of silicic supereruptions from the Afro-Arabian large igneous province
Source: Nat Commun. 2021 Nov 2;12:6299. doi: 10.1038/s41467-021-26468-5 (PMC8563981; doi:10.1038/s41467-021-26468-5)
Supplement: Supplementary file 3 — Description of Additional Supplementary Files [file 41467_2021_26468_MOESM3_ESM.pdf]

Description of Additional Supplementary Files

File Name: Supplementary Data 1
Description: Trace element concentrations and geochronologic data for zircon dated via CA-TIMS.
